# In Vitro Assay of *Paecilomyces lilacinus* Biocontrol Effects on *Fasciola hepatica* Eggs Illustrated in Scanning Electron Micrographs

**Published:** 2017

**Authors:** Faezeh NAJAFI, Sasan REZAIE, Eshrat Beigom KIA, Mahmoud MAHMOUDI, Sadegh KHODAVAISY, Mehdi MOHEBALI, Mohammad Javad GHARAGOZLOU, Mohammad Bagher ROKNI, Gholamreza MOWLAVI

**Affiliations:** 1. Dept. of Parasitology and Mycology, School of Public Health, Tehran University of Medical Sciences, Tehran, Iran; 2. Dept. of Epidemiology and Biostatistics, School of Public Health, Tehran University of Medical Sciences, Tehran, Iran; 3. Center for Research of Endemic Parasites of Iran (CREPI), Tehran University of Medical Sciences, Tehran, Iran; 4. Dept. of Pathobiology, Faculty of Veterinary Medicine, University of Tehran, Tehran, Iran

**Keywords:** *Fasciola hepatica*, *Paecilomyces lilacinus*, Scanning electron micrographs

## Abstract

**Background:**

Fascioliasis is a zoonotic disease caused by the liver fluke *Fasciola hepatica*. Drug resistance, high costs of treatment and economic losses in meat production have emerged the need of alternative control measures into consideration. The aim of this study was to evaluate the in vitro ovicidal activity of *Paecilomyces lilacinus* fungus on *F. hepatica* eggs.

**Methods:**

*P. lilacinus* isolated from the soil of natural environment was challenged on *F. hepatica* eggs to observe the bio control effect of nematophagous fungi on trematode helminth eggs. The study was conducted in Tehran University of Medical Sciences, in 2015. Within 21 d of experiment, destructive effects exhibited on the eggshells were investigated using optical and Scanning Electron Microscopy.

**Results:**

The effective role of *P. lilacinus* on damaging the eggs of *F. hepatica* was noticed.

**Conclusion:**

This finding is promising for advantageous use of nematophagus fungi as a natural constituent in hyper endemic areas for certain helminthic infections like fascioliasis with diverse kinds of herbivores as egg passer hosts.

## Introduction

Fascioliasis caused by liver flukes *Fasciola hepatica* and *F. gigantica* is prevalent in more than 51 countries with 2.4–17 million human occurrences globally (1). Ninety-one million people are globally at the risk of the infection (2). Negative effects of fascioliasis on livestock industry are economically significant (3). A highest prevalence rate of the infection in humans in Bolivia and Peru is reported (4). In Iran, fascioliasis has been known endemic since decades ago emphasizing on two epidemics in 1989 and 1999 in Guilan Province, Caspian Sea littoral, northern Iran which caused 7000 and 10000 human infections, respectively (5). Between the years 1999 to 2002 in the neighboring province, Mazandaran, 107 human fascioliasis was reported (6).

Concerning the huge number of infected livestock worldwide, anthelminthic therapy seems essential in ongoing veterinary programs targeting to reduce the infection in domestic ruminants and transmission control in human population. Regarding the needs to achieve a safer treatment to drug resistance issue (7), enough consideration has been paid nowadays. Accordingly, the biological behavior of nematophagus fungi in damaging process of helminth eggs has been focused and successfully experimented so far (8). Destructive effects of these fungi has been described and illustrated in three types as effect type 1, effect type 2 and the effect type 3, using light and scanning electron micrographs (9).

In the present study, *Paecilomyces lilacinus* isolated from natural environment was experimentally challenged on *F. hepatica eggs*. Since the light microscope cannot exhibit well the details of the mycelia penetration process on the eggshells, Scanning Electron Microscopy (SEM), was employed to prove the effects herein.

## Material and Methods

### Soil sampling and fungi isolation

Three hundred soil samples were collected from 11 different geographical localities of Pakdasht, Damavand, Farahzad, Vanak village and Kan in Tehran Province, Sanandaj and Hamadan in the west, Gilan and Mazandaran in Caspian Sea in the north, Qom and Isfahan province in central Iran in 2014–15. Parks, husbandries and gardens have been already chosen as sampling places in present research. In each locality, 50 gr of soil were collected from the surface to 1.5 cm depth, labeled, kept in zip kips and were stored at room temperature. From the supernatant of the concentrated samples, 200 μl were cultured on 2% water-agar, with two replicates for each sample. According to similar experiment, one ml of distilled water containing of 400 *Rhabditis sp.* larvae was added into the culture media as baiting. Careful observation on cultured plates was continued for two months (10).

For identification of the grown colonies, the abovementioned larvae with attracted mycelia were transferred into potato dextrose agar (PDA) media. Grown strains were preliminarily identified as the species level based by colony morphology and microscopic characteristics and confirmed by partial sequencing of the b-tubulin gene fragment using the primers Bt2a (5′-GGT AAC CAA ATC GGT GCT TTC-3′) and Bt2b (5′- ACC CTC AGT GTA GTG ACC CTT GGC-3′). The PCR setup and programme were followed as described before (11). Prior to the experiment the nematophagus fungus, *Arthrobotrys oligospora,* (CBS 251.82) was cultured by the same described pattern as control sample.

### In vitro assay

Isolated *P. lilacinus* were cultured on PDA media for 10 d stored in 25 °C. The suspension from the grown colonies was prepared and cultured in 2% Water-agar for 10 d in the same temperature. By the end of the 10^th^ day, 500 *F. hepatica* eggs obtained from the slaughterhouse of Tehran, Iran**,** were washed in sterile distilled water and added to the culture media (12). The same process was continued for control plates without fungi. Eighteen plates were cultured for each group. From the day 7^th^, 14^th^ and 21^st^, 100 eggs from each plate were picked up and were carefully inspected under the microscope for observing the effects (X40). Three mentioned types of effects: type 1 (adhering the hyphae to eggshell without morphological alterations), type 2 (morphological alteration on the eggshell and embryo without hyphal penetration) and type 3 (penetration of the hyphal into the eggs and morphological alterations on the eggshell and embryo) described earlier (13), were comparatively investigated. Data from each interval set were analyzed by Friedman nonparametric test at the 5% levels of significance.

## Results

**Fig. 1:**
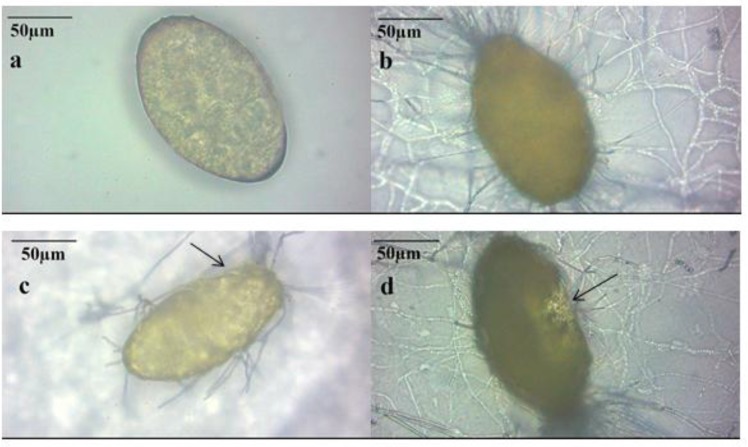
Destructive process observed under the optical microscope (40 ×), (**a)** Intact *F. hepatica* egg in control group, (**b)** Adhering of the eggshell by *P. lilacinus* hyphae, representing effect type 1, (**c)** The beginning of destructive process on the egg by *P. lilacinus* hyphae, representing effect type 2 (black arrow), (**d)** *P. lilacinus* hyphae merged into the egg showing destructive effect*,* representing of effect type3 (black arrow)

Out of all soil samples, *P. lilacinus* harboring nematophagus fungi was significantly recovered from three localities in northwestern vicinity of Tehran. Sequential destructive effects on *F. hepatica* eggs observed under the optical microscope are shown in Fig. 1, comparing with the intact egg in control group.

**Fig. 2:**
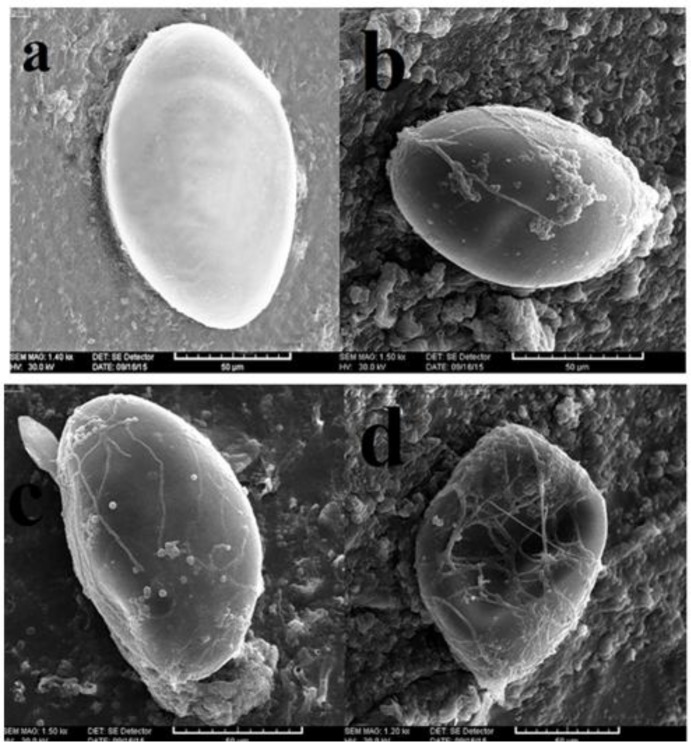
Scanning Electron Micrographs showing the entire process of damaging the eggs of *F. hepatica* comparing with the unchallenged egg in control group, (**a)** Intact *F. hepatica* egg in control group, (**b, c)** Hyphae of *P. lilacinus* adhered to the shell, (**d)** *P. lilacinus* hyphae merged into the egg showing destructive effect

The details of affected eggs in comparison with control group were indicated by SEM technique, (Fig. 2). During the 7^th^, 14^th^ and 21^st^ day of the experiment, the entire steps of attachment towards the final damage of the eggs were clearly detected. According to Freidman, statistical method significant differences of all three types in every 3 d were concluded. In 7^th^ day, in 40% of exposed *F. hepatica* eggs, the effect type 1 demonstrating the mycelial embedding process was seen. In 21^st^ day, the effect type 2 was the most dominant effect, regarding two other types. The effect type 3 was observed in every 3 d with the highest level in 21^st^ day. Different effects during the time schedule of the experiment are shown in Table 1.

**Table 1:** Percentages for the ovicidal activity of *P. lilacinus* on eggs of *F. hepatica* on the 7^th^, 14^th^ and 21^st^ days of interaction

**Isolate**	**Effect type 1**	**Effect type 2**	**Effect type 3**
7^th^ days of interaction			
*Paecilomyces lilacinus*	40.6	30.8	20.2
Control	0	0	0
14^th^ days of interaction			
*Paecilomyces lilacinus*	32.6	35.3	22.3
Control	0	0	0
21^st^ days of interaction			
*Paecilomyces lilacinus*	30	38.3	24.6
Control	0	0	0

Effect type 1: Adhering the hyphae to eggshell without morphological alterations,

Effect type 2: Morphological alterations on the eggshell and embryo without hyphal penetration,

Effect type 3: Penetration of the hyphal into the eggs and morphological alteration on the eggs

## Discussion

In present experiment, the biological capability of nematophagous fungus *P. lilacinus* in *F. hepatica* eggs breaking down from the chain of development were successfully concluded. Attachment of mycelial to dispersed parasite eggs in the natural environment is a promising process in damaging the trapped eggs during the fungi-feeding course on parasite particles. The obtained results have been previously indicated in some studies in the past.

Previous experiments have exhibited the biological destructive effect of nematophagus fungus, *P. lilacinus* on the eggs of cestodes and nematodes successfully (8, 14). The biological effect on the eggs of *Toxocara canis* has been described earlier (14). Destructive effects of the fungus *P. lilacinus* were similarly observed in reduction of onchosphere viability of *Taenia hydatigena* eggs (15).

According to previous experiments (14) and our observation, a successful challenge of nematophagus fungi on targeted eggs can be described, in destroying the eggs. To elaborate the details of these predatory process researchers have categorized the steps of effects in three types during a course of 21-day experiment, as we did. This pattern of effects type 1, 2 and 3 in the 10^th^ day post experiment for instance, has shown as 52.5%, 21% and 25.5% for *T*. *saginata* eggs and *P. lilacinus* respectively (16). Although a similar result by challenging of *Pochonia chlamydosporia* on *F. hepatica* eggs have been observed earlier, using optical microscope (17), destructive effects on these eggs by *P. lilacinus* was clearly illustrated by SEM in the present study (Fig. 2).

**Fig. 3:**
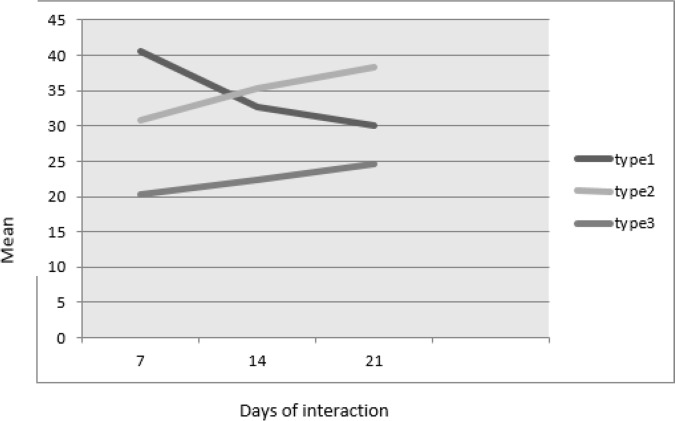
Means of the ovicidal activity of *Paecilomyces lilacinus* on eggs of *Fasciola hepatica* on the 7^th^, 14^th^ and 21^st^ days of interaction

To some extent, pictures taken by optical microscope (Fig. 1: b, c, & d) illustrate the mentioned three types of effects (type 1, 2, 3) concerning control samples (Fig. 1: a). Conclusively, SEM technique, however, exhibited the course of damage more evidently herein, although a firm differentiation between the type 2 and 3 seems controversial (Fig. 2: b, c & d). Meanwhile, Freidman statistical analysis has supported the significant differences of all three types in every 3 days. In addition, during the experiment schedule the effects type 1 was gradually decreased while the effects type 2 and 3 were constituted instead (Fig. 3).

In this study, the essential role of time duration in fungi and parasite eggs interaction indicated that, the longer exposure time might lead to more ovicidal activity by the fungi as has been observed by others (18). A given fungi can be regarded ovicidal when effect type 3 is observable during the entire course of experiment in 7^th^, 14^th^, and 21^st^ days (19), as present findings indicated. From the other points of views, preferential fungal attack assumed to reduce the number of eggs in some archeological sites (20), indicates the possible role of *P. lilacinus* as an egg parasitic fungus in the nature regarded valuable worldwide. Biologically, the mechanism of this confrontation led to destruction of parasites egg in the nature can be explained in mechanical and enzymatic actions of the fungi during the feeding process on organic materials (21). Consequent to fungi attachment on feeding items, hypha apical growth may cause the release of enzymes facilitating the consumption process (22). The fungus, *P. lilacinus* through releasing the protease enzyme (23), against the existed protein in the outer shell of *F. hepatica* eggs (24) may take advantage to destroy the eggs. As the figures presented herein, illustrate a defined types of effects 1, 2 and 3 observed by the light microscope, but our current experiment describes an impartible series of effects by SEM. However, fungi parasitism behavior can explain the extending of feeding activity towards the complete damage of the eggs as trapped baits.

## Conclusion

Dividing the process of destruction to three defined types of effects should be reconsidered as the fungi start the eggs damages, from the attachment step, up to entire round of consumption. The initial confrontation between the fungi and the subjected eggs will trigger a continual non-stoppable process that might be resulted to a complete distraction of the eggs.
